# Comparison of integrated versus parallel continuous renal replacement therapy combined with veno-venous extracorporeal membrane oxygenation in patients with COVID-19 ARDS

**DOI:** 10.1186/s12871-024-02818-w

**Published:** 2025-01-16

**Authors:** Kristina Schönfelder, Felix Helmenstein, Frank Herbstreit, Johanna Reinold, Andreas Kribben, Michael Jahn, Justa Friebus-Kardash

**Affiliations:** 1 Department of Nephrology, University of Duisburg-Essen, University Hospital Essen, Essen, Germany; 2 Department of Anaesthesiology, University of Duisburg-Essen, University Hospital Essen, Essen, Germany; 3 Department of Nephrology, University Hospital Essen, University of Duisburg-Essen, Hufelandstr. 55, 45147 Essen, Germany

**Keywords:** Extracorporeal membrane oxygenation (ECMO), Continuous renal replacement therapy (CRRT), Device combination, COVID 19, ARDS, Integrated connection, Parallel connection, Medical devices, Safety

## Abstract

**Introduction:**

Acute kidney injury (AKI) is a common complication of acute respiratory distress syndrome (ARDS) and multiple organ dysfunction syndrome (MODS) in patients receiving extracorporeal membrane oxygenation (ECMO) support, leading to requirement of continuous renal replacement therapy (CRRT) in 70% of ECMO patients. Parallel arrangement of CRRT and ECMO circuits is common in adult patients. However, CRRT may also be integrated directly into the ECMO circuit. This study compares the safety of both approaches.

**Methods:**

This retrospective analysis included 105 patients treated with continuous veno-venous haemodiafiltration and veno-venous ECMO (Cardiohelp©) for COVID-19-induced ARDS between April 2020 and December 2021. Of these, 48 patients received a parallel connected CRRT running independently from ECMO (parallel approach), while in 57 patients, CRRT was integrated into the ECMO circuit (integrated approach) by connecting the CRRT access line to the post-oxygenator port and the CRRT return line to the pre-oxygenator position. Local protocol for risk assessment of this device combination mandated a maximum return line pressure below 250 mmHg in the CRRT system.

**Results:**

At CRRT initiation, the integrated group had significantly higher median pressures in CRRT lines compared to the parallel approach group (access line 110 mmHg vs. -25 mmHg, return line 170 mmHg vs. 50 mmHg; *p* < 0.01). However, median transmembrane pressures were similar between both groups (20 mmHg vs. 20 mmHg, *p* = 0.16). In-hospital mortality (*p* = 0.99), catheter associated infections (*p* = 0.47), bacteraemia (*p* = 0.96), filter clotting (*p* = 0.58) and unplanned CRRT system changes (*p* = 0.45) within the first 72 h of CRRT were comparable between both groups. The integrated group exhibited higher rates of bleeding events (37% vs. 23%; *p* = 0.08). Thromboembolism occurred in four cases in the integrated group, while one pneumothorax was observed in the parallel group. No cases of air embolism, device associated haemolysis or blood leakage was documented.

**Conclusions:**

Despite higher pressures in CRRT lines, the integrated approach provided comparable safety to the parallel approach. In case of hygienically challenging settings (such as the COVID-19 pandemic), the minimization of extracorporeal accesses and the streamlining of alarm management are decisive factors in providing intensive care medicine. Therefore, the integrated configuration of CRRT into the ECMO circuit can be advantageous in daily intensive care medicine.

**Supplementary Information:**

The online version contains supplementary material available at 10.1186/s12871-024-02818-w.

## Background

Acute kidney injury (AKI) is common in critically ill patients receiving extracorporeal membrane oxygenation (ECMO) as a support for life-threatening cardiac or respiratory insufficiencies (1–2). Up to 70% of adult ECMO patients develop AKI requiring continuous renal replacement therapy (CRRT) to manage fluid overload and metabolic imbalances (3–4). The combination of CRRT with ECMO has been adopted in most ECMO centres [2]. However, no standard method has been recommended thus far [5]. The choice of CRRT delivery approach to ECMO patients often depends on local expertise and institutional protocols (2–3). The classic way for the simultaneous application of CRRT and ECMO is the parallel approach, which is widely used in adult patients and requires a dialysis catheter (5–6). Establishing separate vascular access for independent delivery of CRRT and ECMO can be challenging because ECMO occupies two vascular sites (2–3). Inserting a central venous dialysis catheter in a patient receiving high doses of anticoagulation due to ECMO could increase the risk of bleeding or vascular damage [3–6]. Other complications associated with additional vascular access are thrombosis and infectious (6–7). Otherwise, high blood volume circulating in parallel connected CRRT and ECMO systems could provoke haemodynamic instability [6]. However, with the parallel approach, the standard CRRT prescription can be applied and exchanged by a bedside nurse without direct involvement of an ECMO specialist (2–3, 5, 7). Independent running of CRRT from ECMO allows no interferences of CRRT system with systemic or ECMO haemodynamics [3].

An alternative technique for combining CRRT with ECMO is an integrated approach, which has become increasingly popular in recent years (2–3, 5, 8). Nevertheless, the approach of connecting the CRRT lines directly to the ECMO circuit remains poorly characterised, and exposure of the CRRT device to abnormally high pressures is the main challenge [3, 8]. Exceeding pressure limits carries the risk of haemolysis, microembolisation and flow turbulence (2–3, 9–10). In addition, high pressures and large pressure variations in the CRRT system could trigger pressure alarms, leading to repeated CRRT pump stops, resulting in clots in the CRRT circuit and reduced dialysis dose (2–3, 9–10). However, the integrated delivery of CRRT with ECMO may also offer advantages, including precise control of blood flow, simplified alarm management and minimisation of vascular access (2–3).

In our retrospective observational study, we aimed to compare the two alternative CRRT delivery options in patients on veno-venous ECMO in terms of safety aspects and adverse events.

## Methods

### Study population

We analysed 105 ECMO patients who underwent CRRT in the intensive care unit of the University Hospital Essen between April 2020 and December 2021. We included all adult ECMO patients (≥ 18 years old) who required veno-venous ECMO for the underlying diagnosis of ARDS due to COVID-19 pneumonia and received CRRT because of AKI and fluid overload. The retrospective analysis was reviewed and approved by the local ethics committee of the University Duisburg-Essen (21-9897-BO).

Data related to general demographics, prognostic intensive care scores, mortality, adverse outcomes, complications, laboratory values and information on ECMO and CRRT settings were collected from electronic medical record reviews. Laboratory variables were determined on a daily basis using standard clinical chemistry tests in our Institute of Laboratory Medicine. For the retrospective analysis, all laboratory values were obtained at initiation of CRRT treatment, on subsequent seven days and after completion of CRRT treatment. For our analysis, clinically evident haemolysis was considered when it was documented in the ICU medical records and defined in the clinical context by the attending intensivists using various laboratory constellations such as normocytic normochromic anaemia, thrombocytopenia, elevated bilirubin and lactate dehydrogenase levels, loss of haptoglobin, detection of fragmentocytes or free haemoglobin.

### ECMO and CRRT equipment

ECMO was conducted using the Cardiohelp© system (Maquet Cardiopulmonary GmbH, Rastatt, Germany). ECMO was driven by a centrifugal pump with a heparinised circuit and an oxygenator, using either the HLS set Bioline or the HIT set with a softline coating for patients with heparin-induced thrombocytopenia (HLS set advanced 5.0, HLS set advanced 7.0, Maquet Cardiopulmonary GmbH, Rastatt, Germany). ECMO circuits were inserted using cannulae of varying sizes, ranging from 15 to 25 Fr, depending on the location (19 Fr for the internal jugular vein and 20 to 25 Fr for the femoral vein). Systemic anticoagulation for ECMO was achieved through the administration of unfractionated heparin (first-line) or argatroban with monitoring by serum aPTT.

CRRT was conducted using a multifiltrate machine (Fresenius SE & Co. KGaA, Bad Homburg, Germany), comprising an extracorporeal circuit with a high-flux dialyser F60S (Fresenius, Medical Care AG, Bad Homburg, Germany), haemodiafiltration lines (Meise, Schalksmuehle, Germany), and a blood line extension system (VMP, Iserlohn, Germany). The entire CRRT circuit was exchanged regularly every 72 h. The blood flow rate was set at 100 mL/min. Systemic anticoagulation with unfractionated heparin or argatroban was applied to all patients. In most of the cases additional regional anticoagulation of CRRT circuit was performed using citrate.

### Settings in the integrated approach group (device combination of EMCO and CRRT)

In accordance with previous studies from large referral ECMO centres [3, 11–13], we used the connection of the CRRT access line to the post-oxygenator port and the CRRT return line to the pre-oxygenator port of veno-venous ECMO using three-way taps. The post-pump section of the ECMO circuit is a positive pressure section that develops pressures between 100 and 500 mmHg [3]. The use of the post-pump position for CRRT means that the blood circulates between the centrifugal pump and the oxygenator [3, 11]. The pressure in the post-pump, pre-oxygenator region is usually 30–150 mmHg higher than in the post-pump, post-oxygenator part [11, 14]. Thus, as the least invasive method of working with reduced pressures, we connected the CRRT access line to the post-oxygenator segment of ECMO and the CRRT return line to the post-pump, pre-oxygenator region [3]. The access line pressure alarm range of the multifiltrate dialysis machine used in our study is -280 to 300 mmHg, while the return line pressure alarm range is -80 to 500 mmHg. The pressure between the filter connection and the filter pump, corresponding to the dialysate pressure of the multifiltrate machine, should not exceed 300 mmHg. A limit of 300 mmHg was set as the acceptable maximum pressure level in the return line in our treatment protocol. If this was exceeded, the patient would have been switched to a parallel approach.

### Statistical analysis

The data are expressed as medians and interquartile ranges for continuous variables and as numbers with percentages for dichotomous variables. A comparison of the continuous variables between the two groups with different CRRT configurations (parallel and integrated approach) was conducted by using the Mann-Whitney test. The categorical data between the groups were analysed using the two-tailed chi-square test. To compare pre- and post-treatment values, the Wilcoxon test was performed. Kaplan-Meier survival curves were created and evaluated using the log-rank test. A *p*-value of less than 0.05 was considered statistically significant.

Data analysis was conducted using GraphPad Prism version 6 (GraphPad Software, Inc., La Jolla, CA, USA), IBM SPSS Statistics version 23 (IBM Corp., Armonk, NY, USA) and R (R Core Team 2017, Vienna, Austria).

## Results

### Baseline patient characteristics and extracorporeal settings

Table 1 summarises the main demographic characteristics, laboratory values, ECMO, and CRRT settings at the commencement of the combined organ support.

**Table 1:** Patient’s characteristics at start of combined CRRT and ECMO support

Variable	All *n* = 105	Integrated approach group *n* = 57	Parallel approach group *n* = 48	RR (CI)	*p* value
Age, median (IQR)	55 (47, 60)	53 (47, 59)	56 (45, 62)		0.85
Females, (%)	28 (26)	17 (29)	11 (22)	1.3 (0.7–2.5)	0.43
CRRT citrate anticoagulation + systemic ECMO anticoagulation (%)	100 (95)	56 (98)	44 (91)	1.1 (1.0-1.2)	0.11
Systemic ECMO anticoagulation (%)	5 (4)	1 (2)	4 (8)	0.2 (0.0-1.4)	0.11
**Pre-existing comorbidities**					
Cardiovascular diseases, (%)	59 (56)	34 (60)	25 (52)	1.1 (0.8–1.6)	0.43
Pulmonal diseases, (%)	16 (15)	9 (16)	7 (15)	1.1 (0.5–2.6)	0.86
Kidney diseases, (%)	10 (9)	7 (12)	3 (6)	2.0 (0.6–6.9)	0.28
Disease of central venous system, (%)	14 (13)	7 (12)	7 (14)	0.8 (0.3–2.2)	0.73
Diabetes mellitus (type I-II), (%)	25 (24)	12 (21)	13 (27)	0.7 (0.4–1.5)	0.47
Malignancies, (%)	6 (6)	4 (7)	2 (4)	1.7 (0.4–7.7)	0.53
Diseases of gastrointestinal tract, (%)	21 (20)	12 (21)	9 (19)	1.1 (0.5–2.4)	0.77
**Laboratory values at baseline**					
Leukocytes (/nL), median (IQR)	15.3 (10.3, 21.0)	16.9 (9.6, 24.7)	14.1 (10.8, 20.6)		0.76
Haemoglobin (g/dL), median (IQR)	8.7 (7.9, 9.5)	8.8 (8.0, 9.6)	8.7 (7.8, 9.4)		0.65
Platelets (/nL), median (IQR)	166 (117, 260)	202 (122, 263)	150 (113, 259)		0.27
INR, median (IQR)	1.1 (1.0, 1.2)	1.1 (1.0, 1.3)	1.1 (1.0, 1.2)		0.53
aPTT (sec), median (IQR)	43 (32, 54)	42 (30, 54)	43 (33, 54)		0.68
Fibrinogen (mg/dL), median (IQR)	580 (416, 733)	621 (435, 749)	557 (389, 695)		0.26
Serum creatinine (mg/dL), median (IQR)	1.9 (1.2, 2.9)	1.8 (1.1, 3.2)	2.1 (1.1, 3.2)		0.40
Blood urea nitrogen (mg/dL), median (IQR)	58.3 (40.5, 80.4)	54.7 (40.4, 80.3)	61.5 (40.6, 83.1)		0.67
eGFR (mL/min/1.73 m²), median (IQR)	38 (24, 60)	42 (26, 60)	37 (21, 60)		0.64
Bilirubin (mg/dL), median (IQR)	1.2 (0.8, 2.3)	1.1 (0.7, 2.1)	1.5 (0.8, 3.1)		0.11
Myoglobin (µg/L), median (IQR)	851 (297, 2601)	743 (296, 1959)	963 (297, 3443)		0.52
LDH (U/L), median (IQR)	578 (452, 1073)	540 (453, 740)	610 (452, 868)		0.54
C-reactive protein (mg/dL), median (IQR)	22 (13, 29)	20 (11, 28)	24 (16, 30)		0.08
Procalcitonin (ng/mL), median (IQR)	2.9 (1.0, 9.3)	2.4 (0.8, 8.0)	3.2 (1.1, 10.5)		0.27
**CRRT settings at baseline**					
Blood flow (mL/min), median (IQR)	100 (100, 100)	100 (100, 100)	100 (100, 100)		0.47
Access line pressure (mmHg), median (IQR)	65 (-30, 120)	110 (58, 150)	-25 (-38, 55)		**< 0.01**
Return line pressure (mmHg), median (IQR)	130 (48, 180)	170 (119, 200)	50 (23, 138)		**< 0.01**
TMP (mmHg), median (IQR)	20 (10, 20)	20 (10, 20)	20 (10, 20)		0.17
Ultrafiltration rate (mL/h), median (IQR)	400 (300, 400)	400 (300, 413)	400 (300, 400)		0.35
Dialysate flow (mL/h), median (IQR)	1000 (1000, 1500)	1000 (1000, 1500)	1000 (1000, 1500)		0.78
**ECMO settings at baseline**					
ECMO blood flow (L/min), median (IQR)	4.4 (3.6, 5.3)	4.4 (3.6, 5.0)	4.6 (3.7, 5.8)		0.29
Return pressure (mmHg), median (IQR)	155 (124, 205)	151 (120, 187)	167 (132, 208)		0.13
Drainage pressure (mmHg), median (IQR)	-80 (-108, -52)	-81 (-103, -54)	-80 (-124, -44)		0.87

Activated partial thromboplastin time, aPTT; confidence interval, CI; continuous renal replacement therapy, CRRT; extracorporeal membrane oxygenation, ECMO; estimated glomerular filtration rate – MDRD formula, eGFR; international normalized ratio, INR; lactate dehydrogenase, LDH; liter, L; Transmembrane pressure, TMP; relative risk, RR; unit, U.

Two different CRRT delivery strategies for ECMO patients were compared. Forty-eight patients received separate ECMO and CRRT circuits as parallel running systems (PAG - parallel approach group), while 57 patients had CRRT integrated into the ECMO circuit (IAG - integrated approach group) (Table 1). The ECMO patients in the two groups did not differ significantly in most demographic characteristics. Risk stratification revealed comparable median SAPS II (IAG 73 vs. PAG 75, *p* = 0.57) and SOFA (IAG 14 vs. PAG 15, *p* = 0.15) scores at baseline (Table 2). There were no significant differences in baseline laboratory values between the two groups (Table 1). The ECMO settings were also similar in both groups (median blood flow IAG 4.4 L/min vs. PAG 4.6 L/min, *p* = 0.29) (Table 1). CRRT settings were comparable between the two groups, with the exception of the pressure values in access and return lines, which were significantly higher in IAG than in PAG (median access line pressure IAG 110 mmHg vs. PAG − 25 mmHg, *p* < 0.0001; median return line pressure IAG 170 mmHg vs. PAG 50 mmHg, *p* < 0.0001).

**Table 2:** Outcome between CRRT circuit integrated into the ECMO circuit and parallel running CRRT and ECMO

Variable	All, *n* = 105	Integrated approach group, *n* = 57	Parallel approach group, *n* = 48	RR (CI)	*p* value
In-hospital mortality, (%)	92 (88)	50 (89)	42 (89)	1.0 (0.9–1.2)	0.97
SAPS II score at therapy start, median (IQR)	74 (69, 79)	73 (69, 78)	75 (68, 80)		0.57
SOFA score at therapy start, median (IQR)	15 (13, 16)	14 (13, 16)	15 (15, 16)		0.15
Patients decannulated from ECMO, (%)	17 (16)	8 (14)	9 (19)	0.8 (0.3–1.8)	0.51
ECMO duration in decannulated patients (days), median (IQR)	17.6 (7.7, 26.8)	22.6 (8.2, 33.6)	11.0 (5.2, 20.1)		0.19
CRRT in decannulated patients (days), median (IQR)	23.5 (12.3, 32.1)	23.6 (12.2, 36.9)	23.5 (12.5, 29.4)		0.81
Stay at intensive care unit (days), median (IQR)	16.5 (11.1, 25.8)	18.5 (12.4, 26.6)	14 (9, 24)		0.23
Transfer from intensive care unit, (%)	7 (6)	3 (5)	4 (8)	0.6 (0.2–2.4)	0.53
Time alive (days), median (IQR)	8.5 (4.8,16.3)	9.2 (5.7,16.1)	6.7 (2.9,16.8)		0.18
Recovery from AKI, (%)	11 (11)	5 (9)	6 (13)	0.7 (0.2–2.1)	0.53
CKDG5D among survivors, (%)	5/11 (45)	3/6 (50)	2/5 (40)	1.3 (0.4–4.9)	0.74
**Complications**					
Bleedings, (%)	33 (31)	22 (39)	11 (23)	1.7 (0.9–3.1)	0.08
Otorhinolaryngological area, (%)	14 (13)	9 (16)	5 (10)	1.5 (0.6–4.1)	0.42
Bronchopulmonal, (%)	5 (5)	5 (9)	0 (0)	∞ (1.1- ∞)	**0.04**
Gynaecological, (%)	2 (2)	1 (2)	1 (2)	0.8 (0.1-8.0)	0.90
Gastrointestinal, (%)	10 (9)	6 (10)	4 (8)	1.2 (0.4-4.0)	0.70
Brain haemorrhages, (%)	5 (5)	2 (3)	3 (6)	0.6 (0.1–2.7)	0.51
Musculoskeletal, (%)	3 (3)	3 (5)	0 (0)	∞ (0.7-∞)	0.10
Bleeding at the ECMO site, (%)	3 (3)	2 (3)	1 (2)	1.6 (0.2–12.7)	0.66
Thromboembolism, (%)	4 (4)	4 (7)	0 (0)	∞ (0.9-∞)	0.06
Heparin induced thrombocytopenia II, (%)	1 (1)	0 (0)	1 (2)	0.0 (0.0-3.2)	0.27
Central venous catheter associated infections, (%)	16 (15)	10 (17)	6 (12)	1.4 (0.6–3.5)	0.47
Bacteraemia, (%)	61 (58)	33 (58)	28 (58)	0.9 (0.7–1.4)	0.96
Filter clotting events, (%)	54 (51)	30 (53)	24 (50)	1.0 (0.7–1.5)	0.78
Filter clotting events within the first 72 h of CRRT, (%)	29 (27)	17 (30)	12 (25)	1.2 (0.6–2.2)	0.58
CRRT circuit change within the first 72 h of CRRT, (%)	44 (42)	22 (39)	22 (46)	0.8 (0.5–1.3)	0.45

AKI, acute kidney injury; confidence interval, CI; continuous renal replacement therapy, CKDG5D, end-stage chronic kidney disease requiring dialysis; CRRT; extracorporeal membrane oxygenation, ECMO; relative risk, RR; Simplified Acute Physiology Score II, SAPS II; Sequential Organ Failure Assessment, SOFA.

### Comparison of in-hospital mortality and CRRT complications

**Fig. 1:**
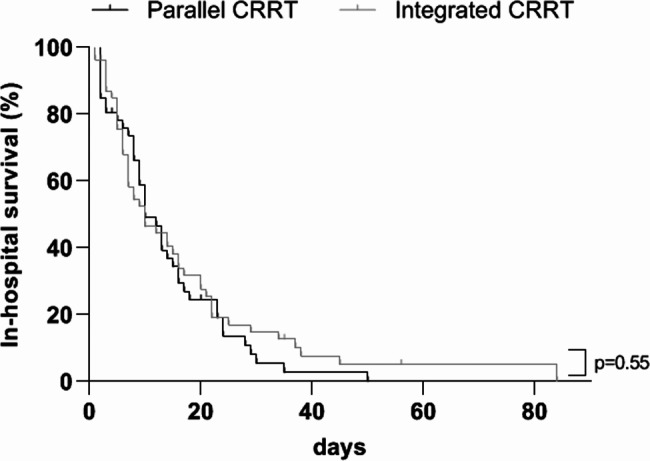
Comparison of in-hospital mortality rates in patients receiving integrated (*n* = 57) and parallel (*n* = 48) configurations of CRRT and ECMO Continuous renal replacement therapy, CRRT; extracorporeal membrane oxygenation, ECMO

In-hospital mortality rates were high but comparable between the two groups (89% IAG vs. 89% PAG, *p* = 0.97; Table 2; Fig. 1A). The length of stay in intensive care (IAG 19 days vs. PAG 14 days, *p* = 0.23; Table 2) was similar between the two CRRT configurations. There was no difference in the length of time patients remained alive from the start of either combination of CRRT and ECMO (IAG 9 days vs. PAG 7 days, *p* = 0.18; Table 2). A total of 17 patients survived until decannulation from ECMO support (IAG 8 vs. PAG 9; Table 2). The duration of ECMO support (IAG 23 days vs. PAG 11 days, *p* = 0.19; Table 2) as well as the duration of CRRT (IAG 24 days vs. PAG 24 days, *p* = 0.81; Table 2) did not differ between the two CRRT delivery approaches in this particular subgroup of patients. The duration of CRRT after decannulation was also comparable in both groups (IAG 6.7 days (5.1, 21.0) vs. PAG 13.7 days (6.4, 23.7), *p* = 0.44). Among the 11 total survivors, the median duration of CRRT (IAG 19 days vs. PAG 24 days, *p* = 0.93) and ECMO support (IAG 15 days vs. PAG 8 days, *p* = 0.25) were comparable between parallel and integrated CRRT delivery strategies. No patient in either group was switched from CRRT to intermittent haemodialysis during their current stay. All survivors were transferred to intensive care units in other hospitals for weaning from the ventilator. Post-discharge clinical records showed that five of the eleven survivors remained on dialysis permanently, with no differences between the groups (IAG 3/6, PAG 2/4, *p* = 0.74, Table 2).

The incidence of all bleeding events was slightly increased in the IAG, though this did not reach statistical significance (IAG 39% vs. PAG 23%, *p* = 0.08; Table 2). Further differentiation between sources of bleeding showed a significantly increased proportion of bronchopulmonal bleedings within the IAG (Table 2). Thromboembolism occurred in only a few patients receiving CRRT integrated into the ECMO circuit (Table 2). The frequency of central venous catheter-associated infections or evidence of bacteraemia was similar for both CRRT delivery approaches (Table 2). Other complications related to the integrated connection of CRRT into the ECMO, such as air embolism or leakage in the connecting lines, were not reported in our study cohort. When analysing the typical complications of the parallel configuration of CRRT and ECMO related to the central venous catheter, we found that a pneumothorax developed directly after the insertion of a Shaldon catheter in only one patient, no arterial vessel injuries were observed.

The number of cases of membrane clotting within the first 72 h of CRRT did not differ between the two CRRT delivery strategies, reflecting comparable filter lifespans in both groups (IAG 30% vs. PAG 25%, *p* = 0.58; Table 2). The percentage of ECMO patients undergoing a CRRT circuit change within the first 72 h of CRRT was also similar between the two groups (IAG 39% vs. PAG 46%, *p* = 0.45; Table 2).

### Laboratory values and CRRT and ECMO settings

**Fig. 2:**
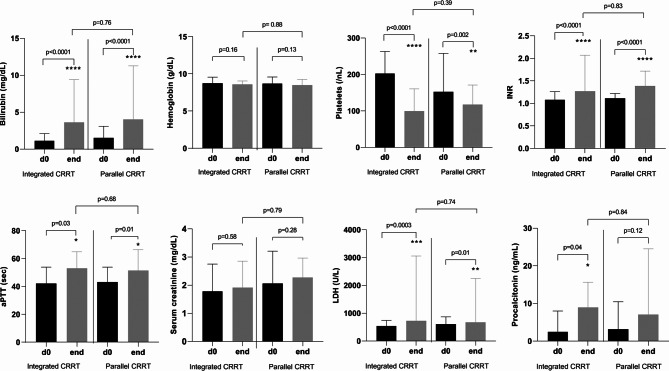
Alterations in laboratory values comparing pre- and post-treatment values in patients with integrated (*n* = 57) versus parallel (*n* = 48) CRRT-ECMO *, *p* = 0.05; **, *p* = 0.01; ****, *p* ≤ 0.0001. Activated partial thromboplastin time, aPTT; continuous renal replacement therapy, CRRT; extracorporeal membrane oxygenation, ECMO; estimated glomerular filtration rate, eGFR; international normalized ratio, INR; lactate dehydrogenase, LDH; liter, L; unit, U

The course of several laboratory values at the beginning and end of CRRT is shown in Fig. 2, depicting that aPTT, LDH, INR, bilirubin and procalcitonin significantly increased, while the platelet counts significantly decreased in both groups. Supplementary Fig. 1 and Supplementary Table 1 provide an overview on changes in laboratory values over the 7-day follow-up period for each CRRT delivery approach.

**Fig. 3:**
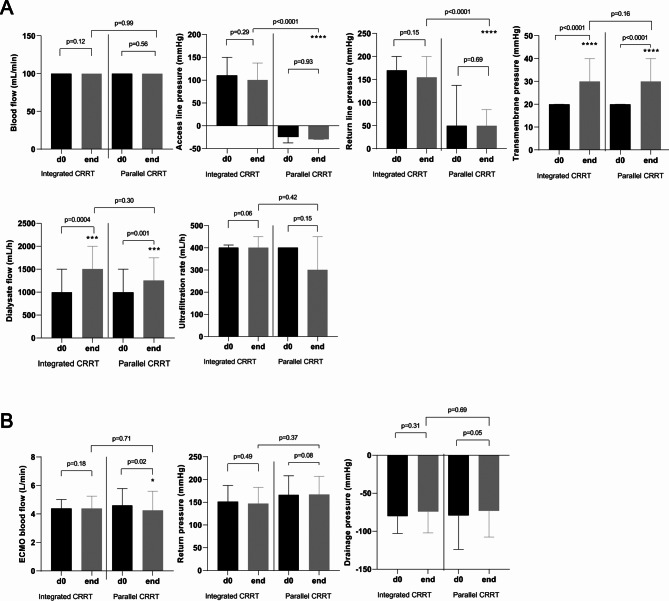
CRRT (**A**) and ECMO settings (**B**) pre- and post-treatment values in patients with integrated (*n* = 57) versus parallel (*n* = 48) CRRT-ECMO *, *p* = 0.05; ***, *p* = 0.001; ****, *p* ≤ 0.0001. Continuous renal replacement therapy, CRRT; extracorporeal membrane oxygenation, ECMO

The evolution of CRRT settings over the maximum follow-up period of 7 days is shown in Supplementary Fig. 2A and Supplementary Table 2. Pressure parameters and blood flow remained stable in both groups (Fig. 3A). In line with our expectations, the median access and return line pressures were significantly higher in the IAG than in the PAG over the whole observation period (Fig. 3A and Supplementary Table 2). The upper pressure limit of 300 mmHg of the CRRT return line was not exceeded in the entire IAG (Fig. 3A).

Furthermore, no relevant variations in ECMO parameters, particularly pressures and blood flows, were observed when comparing within each group and between the two CRRT delivery groups during the dedicated follow-up period (Supplementary Table 3, Fig. 3B and Supplementary Fig. 2B).

With respect to the relationship between the ECMO return pressure levels and the pressure levels in the return line of the CRRT, a positive correlation was found in the IAG, particularly on days 1–3, day 7, and generally at the end of CRRT treatment (Supplementary Fig. 3).

## Discussion

Our retrospective study demonstrated that integrating CRRT into the ECMO circuit in 57 patients had a safety and efficacy profile comparable to that of the independent parallel CRRT and ECMO configuration in 48 patients. There were no significant differences in in-hospital mortality between the two modes of CRRT and ECMO connection, nor did they significantly impact on the length of stay in the intensive care unit. The integrated delivery approach was not associated with a significant increase of complications (e.g., haemolysis, CRRT or ECMO machine failure, CRRT or ECMO tubing leaks, pressure alarms in either extracorporeal system), filter clotting, or the need for CRRT circuit changes. Both approaches showed no differences in the course of the patients’ laboratory values, and CRRT settings remained stable during therapy using the two different connection modes.

To date, there are no definitive guidelines recommending which combination technique should be used in ECMO patients with AKI requiring concomitant CRRT [3]. Any integration of the CRRT circuit directly to the ECMO circuit is not officially approved by the Food and Drug Administration nor by the manufacturers, and clinical practice is primarily based on expert opinion and local experience [3, 6, 15]. Two recent multicenter international surveys reported that up to 60% of centres used an integrated in-series approach, while only 40% of units used the parallel connection technique (16–17). Currently, there have been few comparative studies with small sample sizes on this issue [6, 15].

Independent central venous vascular access is required for the parallel conduction of CRRT and ECMO (2–3, 6, 15). Given the systemic heparinisation and prolonged coagulation time in ECMO patients, it has been suggested that this approach is associated with an increased risk of bleeding, particularly with invasive central venous catheter placement (2–3, 6, 11, 15). However, our analysis did not show a significantly increased incidence of bleeding in the PAG. In fact, there was a slight trend towards a higher frequency of bleeding events in the IAG. In general, catheter site bleeding was rare. The majority of bleeding events were non-serious and occurred in the otorhinolaryngological area. However, all of the few cases of pulmonary bleeding were documented in the IAG. Both groups had comparable platelet counts and coagulation values at baseline, and initial anticoagulation strategies were similar for both approaches. The increase in aPTT and INR during support was observed at similar levels in both groups. However, the IAG experienced a greater decrease in platelet count at the end of therapy than the PAG, which may have predisposed them to observed higher rate of pulmonary bleeding. At the same time, four cases of systemic thromboembolism in our study were also reported only in the IAG, which contrasts with previously reported higher rates of systemic thromboembolism in the parallel use of CRRT and ECMO (2–3, 6, 15, 18). In general, ECMO population is prone to thromboembolism because of the overexpression of cytokines related to the ECMO support, which induces activation of the coagulation cascade [19–21]. In addition, ARDS due to COVID-19 pneumonia is often associated with thromboembolic events driven by thromboinflammation [22]. Endothelial dysfunction, dysregulation of the innate and adaptive immune systems leading to overwhelming production of proinflammatory cytokines, and platelet hyperactivation leading to release of large amounts of chemokines and subsequent activation of the complement system and the coagulation cascade are key pathological responses of thromboinflammatory processes that contribute to thrombus formation during SARS-CoV-2 virus infection [22].

Contrary to the assumption of an increased risk of infections with parallel CRRT connection using an additional central venous catheter, we did not observe any differences in central venous catheter-associated infections or bacteraemia in our study cohort (2–3, 6, 15). Regarding other potential complications of the parallel approach requiring additional vascular access, we observed a pneumothorax in only one patient in the PAG after central venous dialysis catheter insertion, and no vascular injury. The creation of a central venous access is a challenging and potentially limited procedure when two vascular access sites are concurrently utilised by ECMO [3]. In certain cases, the introduction of a third vascular access may be necessary to achieve the required blood supply for the ECMO support [3]. Additionally, a number of anatomical or pathological scenarios (e.g. vascular anomalies, thromboses, skin infections in the vascular access area) can limit the installation of a venous access for an independently running CRRT. In such clinical scenarios, a combination of both extracorporeal procedures will inevitably need to be considered, weighing the risks and benefits. Running the CRRT and ECMO systems independently from one another may also require deeper sedation and patient restraint to reduce CRRT alarms, which may contribute to additional complications and exacerbate patient discomfort [3].

Haemolysis due to shear stress and wall impact forces is frequently observed in ECMO patients [23]. The research group of Chen et al. proposed a higher incidence of haemolysis when using ECMO as a direct venous access for CRRT due to the higher pressure in the CRRT circuit in this setting [24]. In the case of two separate circuits for CRRT and ECMO, there is no interference of ECMO haemodynamics with the CRRT machine, theoretically placing the patients at lower risk of haemolysis (2–3, 6, 15). In our study, suggestive haemolysis parameters such as bilirubin and LDH significantly increased, and platelet counts significantly decreased towards the end of supportive treatment; however, there were no relevant differences between the two groups. Haemoglobin levels did not change during support with either CRRT configuration. Therefore, our results did not confirm the previously reported increase in haemolysis in patients with an integrated CRRT connection.

The studies by de Tymowski et al., Crosswell et al., Raja et al. and Wu et al. described significantly higher filter lifespans in their integrated ECMO and CRRT configuration than in their parallel configuration (11–12, 25–26). The findings of our study indicate no statistically significant differences in filter lifetime between the two groups, regardless of the CRRT connection mode. Following the initiation of CRRT, 40% of patients required a system change before the routine 72-hour change. Filter clots were identified as the reason in 30% of cases across both groups. We speculate that the combination of systemic heparin anticoagulation and local citrate anticoagulation, used in more than 90% of patients in both modes, may have prevented early filter clotting in both groups. Giani et al. described that the combination of unfractionated heparin and citrate led to significantly fewer filter changes than the use of unfractionated heparin alone [27]. Otherwise, both CRRT connection techniques have practical drawbacks that could potentially provoke clotting and shorten the lifespan of dialysis filters. On the one hand, problems with central venous dialysis catheters, including catheter strain due to repositioning or other catheter manipulations, as well as disconnection due to transfer to operating theatres or computerised tomography scans, are common reasons for alarms and iterative stops, which interfere with maintaining stable CRRT blood flows and achieving high ultrafiltration fractions, leading to premature filter clotting (2–3, 6, 15). On the other hand, potential exposure of CRRT access and return lines to inappropriately high pressures that are incompatible with CRRT pressure thresholds in ECMO-integrated CRRT is associated with technical problems and CRRT machine pressure alarms, which additionally result in repeated interruptions of CRRT sessions with subsequent reduction in filter lifespan and dialysis efficacy (2–3, 6, 15).

Post-pump settings were utilised at our centre, with the understanding that recirculation in the dialysis circuit is negligible. In our integrated approach group (IAG), the drainage pressure corresponding to the outflow pressure of the ECMO pre-oxygenator segment did not exceed the maximum range of the venous pressure alarm of 300 mmHg over the 7-day follow-up, contributing to an acceptable frequency of clotting and unplanned CRRT system changes. Transmembrane pressure (TMP) was also maintained within an acceptable range of 20–40 mmHg. The ECMO blood flow in our integrated CRRT connection group was moderate between 4 and 5 L/min, which may have also protected against the development of higher outlet pressures in CRRT lines above the safety threshold of 300 mmHg. The pre-oxygenator pressure range increases with higher ECMO blood flows (maximum ECMO flow is 6 L/min) [13]. The oxygenator traps air bubbles and clots, which is a major advantage of this integrated CRRT configuration chosen in our centre [3, 9]. Accordingly, no cases of air or blood leakage were observed in the IAG of our study. Based on our data and in agreement with previously published work, we think that connecting the CRRT inflow line to the return ECMO line after the oxygenator and the CRRT outflow line to the drainage ECMO line just before the oxygenator is an optimal solution for running CRRT on ECMO. This allows for a well-tolerated venous outlet pressure within the safety range of a maximum of 300 mmHg, thereby not affecting blood flow in the CRRT circuit, and provides an efficient dialysis dose without downtime [3, 11–13]. In general, ECMO blood flow rates are lower in veno-arterial ECMO than in veno-venous ECMO [6]. Therefore, we assume that the aforementioned integrated CRRT connection method to the oxygenator should be particularly recommended for CRRT integration into veno-venous ECMO.

We recognise several limitations of our study, such as its retrospective design, which is prone to selection and information bias. Selection bias may be introduced by the non-randomised choice of the CRRT configuration by the ICU and nephrology teams. For example, it is conceivable that patients at high risk of bleeding or with limited vascular access options were predominantly allocated to the IAG, whereas patients who were intolerant of anticoagulation or prone to thromboembolic events were predominantly allocated to the PAG. Some important laboratory values for the assessment of haemolysis, such as haptoglobin, were either unavailable or lacking in the majority of ECMO patients. In addition, the high mortality rate of 89% in both groups may have introduced a mortality time bias. In other words, the effects of different dialysis configurations are overshadowed by the severity of the disease and its high mortality. The small number of survivors insufficient to detect medium to long-term CRRT configuration-specific effects, as the patients will have died of the disease before. Therefore, a larger number of patients who could be successfully weaned from ECMO would be needed in a future study to provide more robust statistical confirmation of our reported equivalent complication rates between the two dialysis configurations. However, the strengths of our study include the relatively large number of subjects in both CRRT connection groups, recruited over a short period without changes in ECMO and CRRT integration practices, and the high homogeneity of the study population, all of whom were suffering from acute respiratory distress syndrome induced by COVID-19 pneumonia.

## Conclusions

In conclusion, our study showed that the integrated CRTT approach was comparable to conventional parallel CRRT and ECMO in terms of safety. Large prospective randomised comparative trials are needed in order to standardise the integrated CRRT approach. The integration of CRRT into the ECMO circuit is a straightforward method of obtaining circulatory access for in-line CRRT, reducing procedural risks and avoiding the risks associated with the placement of a separate central venous catheter, particularly in ECMO patients with vascular insufficiency. This is even more relevant for patients requiring complex hygiene measures (e.g., COVID-19), as the integrated approach limits alarms related to blood flow and catheter dysfunction of both extracorporeal systems to two access routes. Simplified management of blood flow alarms would be a step toward reducing exposure times for healthcare workers to patients with infectious diseases, decreasing the consumption of equipment (e.g., protective clothing), and thereby reducing the overall cost burden.

## Electronic supplementary material

Below is the link to the electronic supplementary material.

Supplementary Material 1

## Data Availability

The datasets used and analysed during the current study are available from the corresponding author on reasonable request, but are also entirely included in this published article.

## Abbreviations

AKI: Acute Kidney Injury
aPTT: Activated Partial Thromboplastin Time
ARDS: Acute Respiratory Distress Syndrome
AUROC: Area Under the Receiver Operating characteristic Curve
CI: Confidence Interval
CKDG5D: End-stage chronic kidney disease requiring dialysis
COVID: 19-COronaVIrus Disease 2019
CRP: C-Reactive Protein
CRRT: Continuous Renal Replacement Therapy
ECMO: ExtraCorporeal Membrane Oxygenation
eGFR: Estimated Glomerular Filtration Rate
IAG: Integrated Approach Group
INR: International Normalized Ratio
LDH: Lactate DeHydrogenase
PAG: Parallel Approach Group
RR: Relative Risk
SAPS II: Simplified Acute Physiology Score II
SOFA: Sequential Organ Failure Assessment
TMP: TransMembrane Pressure

## References

[1] Brogan TV, Lequier L, Lorusso R, et al. Extracorporeal life support: the ELSO red book. 5th ed. Ann Arbor, MI: Extracorporeal Life Support Organisation; 2017.

[2] Foti L, Villa G, Romagnoli S, Ricci Z. Acute kidney Injury and extracorporeal membrane oxygenation: review on multiple organ support options. Int J Nephrol Renovasc Dis. 2021;14:321–9. 34413667 10.2147/IJNRD.S292893 PMC8370847

[3] Seczyńska B, Królikowski W, Nowak I, Jankowski M, Szułdrzyński K, Szczeklik W. Continuous renal replacement therapy during extracorporeal membrane oxygenation in patients treated in medical intensive care unit: technical considerations – CRRT during ECMO-technical considerations. Ther Apher Dial. 2014;18(6):523–34. 25195931 10.1111/1744-9987.12188

[4] Shum H, Kwan AMC, Chan KC, Yan WW. The use of regional citrate anticoagulation continuous venovenous hemofiltration in extracorporeal membrane oxygenation. ASAIO J. 2014;60(4):413–8. 24727536 10.1097/MAT.0000000000000085

[5] Ostermann M, Connor M Jr, Kashani K. Continuous renal replacement therapy during extracorporeal membrane oxygenation: why,when and how? Curr Opin Crit Care. 2018;24(6):493–503. 30325343 10.1097/MCC.0000000000000559

[6] Zeidman AD. Extracorporeal membrane oxygenation and continuous kidney replacement therapy: Technology and outcomes - A Narrative Review. Adv Chronic Kidney Dis. 2021;28(1):29–36. 34389134 10.1053/j.ackd.2021.04.004

[7] Jacobs R, Honore PM, Spapen HD. Intertwining extracorporeal membrane oxygenation and continuous renal replacement therapy: sense or nonsense? Crit Care. 2015;19(1):145. 25888440 10.1186/s13054-015-0860-6 PMC4373063

[8] Santiago MJ, Sánchez A, López-Herce J, Pérez R, del Castillo J, Urbano J, et al. The use of continuous renal replacement therapy in series with extracorporeal membrane oxygenation. Kidney Int. 2009;76(12):1289–92. 19794394 10.1038/ki.2009.383

[9] MacLaren G, Combes A, Bartlett RH. Contemporary extra-corporeal membrane oxygenation for adult respiratory failure: life support in the new era. Intensive Care Med. 2012;38(2):210–20. 22147116 10.1007/s00134-011-2439-2

[10] Simons AP, Weerwind PW. Re: how to perform a haemodialysis using the arterial and venous lines of an extracorporeal life support. Eur J Cardiothorac Surg. 2011;39(6):1084–5. 20952205 10.1016/j.ejcts.2010.09.010

[11] Wu J, Huang X, Mei Y, Lv J, Li W, Hu D, et al. Impact of connecting methods of continuous renal replacement therapy device on patients underwent extracorporeal membrane oxygenation: a retrospectively observational study. Aust Crit Care. 2023;36(5):695–701. 36610945 10.1016/j.aucc.2022.11.005

[12] Raja M, Leal R, James Doyle J. Continuous renal replacement therapy in patients receiving extracorporeal membrane oxygenation therapy. J Intensive Care Soc. 2023;24(2):227–9. 37260434 10.1177/17511437211067088 PMC10227899

[13] Zhou XL, Chen YH, Wang QY. A new approach combining venoarterial extracorporeal membrane oxygenation and CRRT for adults: a retrospective study. Int J Artif Organs. 2017;40(7):345–9. 28574109 10.5301/ijao.5000597

[14] Sidebotham D, Allen SJ, McGeorge A, Ibbott N, Willcox T. Venovenous extracorporeal membrane oxygenation in adults: practical aspects of circuits, cannulae, and procedures. J Cardiothorac Vasc Anesth. 2012;26(5):893–909. 22503344 10.1053/j.jvca.2012.02.001

[15] Costa AM, Halfwerk F, Wiegmann B, Neidlin M, Arens J. Trends, advantages and disadvantages in combined extracorporeal lung and kidney support from a Technical Point of View. Front Med Technol 2022:4:909990. 10.3389/fmedt.2022.909990 PMC9255675 35800469

[16] Fleming GM, Askenazi DJ, Bridges BC, Cooper DS, Paden ML, Selewski DT, et al. A multicenter international survey of renal supportive therapy during ECMO: the kidney intervention during extracorporeal membrane oxygenation (KIDMO) group. ASAIO J. 2012;58(4):407–14. 22588147 10.1097/MAT.0b013e3182579218 PMC5486948

[17] Thy M, Augustin P, Tran-Dinh A, Montravers P, de Tymowski C. Renal replacement therapy for patients requiring extracorporeal membrane oxygenation: a Multicenter International Survey. Blood Purif. 2022;51(11):899–906. 35390796 10.1159/000522398

[18] Rubin S, Poncet A, Wynckel A, Baehrel B. How to perform a haemodialysis using the arterial and venous lines of an extracorporeal life support. Eur J Cardiothorac Surg. 2010;37(4):967–8. 19914083 10.1016/j.ejcts.2009.10.007

[19] Millar JE, Jonathon P, Fanning JP, McDonald CI, McAuley DF, Fraser JF. The inflammatory response to extracorporeal membrane oxygenation (ECMO): a review of the pathophysiology. Crit Care. 2016;20(1):387. 27890016 10.1186/s13054-016-1570-4 PMC5125043

[20] Chenoweth DE, Cooper SW, Hugli TE, Stewart RW, Blackstone EH, Kirklin JW. Complement activation during cardiopulmonary bypass: evidence for generation of C3a and C5a anaphylatoxins. N Engl J Med. 1981;304(9):497–503. 7453783 10.1056/NEJM198102263040901

[21] Bruins P, Te Velthuis H, Yazdanbakhsh AP, Jansen PG, van Hardevelt FW, de Beaumont EM, et al. Activation of the complement system during and after cardiopulmonary bypass surgery: postsurgery activation involves C-reactive protein and is associated with postoperative arrhythmia. Circulation. 1997;96(10):3542–8. 9396453 10.1161/01.cir.96.10.3542

[22] Higashikuni Y, Liu W, Obana T, Sata M. Pathogenic basis of Thromboinflammation and Endothelial Injury in COVID-19: current findings and therapeutic implications. Int J Mol Sci. 2021;22(21):12081. 34769508 10.3390/ijms222112081 PMC8584434

[23] Mulholland JW, Massey W, Shelton JC. Investigation and quantification of the blood trauma caused by the combined dynamic forces experienced during cardiopulmonary bypass. Perfusion. 2000;15(6):485–94. 11131211 10.1177/026765910001500603

[24] Chen H, Yu R, Yin N, Zhou JX. Combination of extracorporeal membrane oxygenation and continuous renal replacement therapy in critically ill patients: a systematic review. Crit Care. 2014;18(6):675. 25482187 10.1186/s13054-014-0675-x PMC4277651

[25] de Tymowski C, Augustin P, Houissa H, Allou N, Montravers P, Delzongle A, et al. CRRT connected to ECMO: managing high pressures. ASAIO J. 2017;63(1):48–52. 27660903 10.1097/MAT.0000000000000441

[26] Crosswell A, Roodenburg O. Vascular access site influences circuit life in continuous renal replacement therapy. Crit Care Resusc. 2014;16(2):127–30. 24888283

[27] Giani M, Scaravilli V, Stefanini F, Valsecchi G, Rona R, Grasselli G, et al. Continuous renal replacement therapy in venovenous extracorporeal membrane oxygenation: a retrospective study on regional citrate anticoagulation. ASAIO J. 2020;66(3):332–8. 31045918 10.1097/MAT.0000000000001003

