# Supplementary material for: Comparison of integrated versus parallel continuous renal replacement therapy combined with veno-venous extracorporeal membrane oxygenation in patients with COVID-19 ARDS
Source: BMC Anesthesiol. 2025 Jan 16;25:28. doi: 10.1186/s12871-024-02818-w (PMC11737070; doi:10.1186/s12871-024-02818-w)
Supplement: Supplementary file 1 — Supplementary Material 1 [file 12871_2024_2818_MOESM1_ESM.docx]

1. **Supplementary Tables**

**Supplementary Table 1.** Seven-day follow-up of CRRT integrated into ECMO circuit or CRRT and ECMO running in parallel.

| **Day after starting support** | **Integrated approach group n=57** | **Parallel approach group  n=48** | **p value** |
| --- | --- | --- | --- |
| **Day 1 (available values/total group)** | 54/57 | 47/48 |  |
| Leukocytes (/nL), median (IQR) | 14.3 (9.3, 19.0) | 12.7 (10.2, 18.6) | 0.53 |
| Haemoglobin (g/dL), median (IQR) | 8.5 (8.0, 9.1) | 8.4 (7.7, 9.5) | 0.62 |
| Platlets (/nL), median (IQR) | 153 (109, 218) | 131 (89, 201) | 0.32 |
| INR, median (IQR) | 1.1 (1.0, 1.3) | 1.2 (1.1, 1.4) | 0.23 |
| aPTT (sec), median (IQR) | 39.1 (32.1, 49.9) | 45.3 (37.2, 56.6) | **0.04** |
| Fibrinogen (mg/dL), median (IQR) | 519 (364, 667) | 505 (346, 645) | 0.59 |
| Serum creatinine (mg/dL), median (IQR) | 1.9 (1.0, 2.7) | 2.0 (1.1, 3.0) | 0.36 |
| Blood urea nitrogen (mg/dL), median (IQR) | 50.4 (36.8, 67.5) | 54.7 (37.8, 73.2) | 0.42 |
| eGFR (MDRD formula; mL/min/1.73m²), median (IQR) | 39 (24, 60) | 38 (21, 60) | 0.64 |
| Bilirubin (mg/dL), median (IQR) | 1.1 (0.8, 2.2) | 1.9 (0.9, 3.7) | 0.06 |
| Myoglobin (µg/L), median (IQR) | 801 (337, 2211) | 947 (372, 3305) | 0.46 |
| LDH (U/L), median (IQR) | 566 (454, 777) | 567 (430, 727) | 0.91 |
| C-reactive protein (mg/dL), median (IQR) | 19.4 (11.4, 27.6) | 24.7 (14.7, 29.1) | 0.08 |
| Procalcitonin (ng/mL), median (IQR) | 3.7 (1.2, 11.9) | 4.4 (1.5, 13.1) | 0.83 |
| **Day 2 (available values/total group)** | 53/57 | 47/48 |  |
| Leukocytes (/nL), median (IQR) | 14.3 (8.2, 20.2) | 13.6 (10.3, 17.8) | 0.41 |
| Haemoglobin (g/dL), median (IQR) | 8.4 (7.9, 9.1) | 8.5 (7.8, 9.0) | 0.91 |
| Platlets (/nL), median (IQR) | 151 (90, 208) | 128 (79, 193) | 0.44 |
| INR, median (IQR) | 1.1 (1.0, 1.3) | 1.2 (1.1, 1.3) | 0.42 |
| aPTT (sec), median (IQR) | 43.5 (36.9, 58.7) | 45.7 (35.7, 54.3) | 0.78 |
| Fibrinogen (mg/dL), median (IQR) | 486 (363, 661) | 516 (423, 659) | 0.78 |
| Serum creatinine (mg/dL), median (IQR) | 1.5 (1.0, 2.4) | 2.1 (1.0, 2.6) | 0.17 |
| Blood urea nitrogen (mg/dL), median (IQR) | 40.8 (29.4, 59.2) | 45.7 (32.1, 65.5) | 0.34 |
| eGFR (MDRD formula; mL/min/1.73m²), median (IQR) | 46 (30, 60) | 35 (26, 60) | 0.20 |
| Bilirubin (mg/dL), median (IQR) | 1.3 (0.9, 2.6) | 1.7 (1.0, 4.4) | 0.09 |
| Myoglobin (µg/L), median (IQR) | 918 (418, 3555) | 1251 (695, 3358) | 0.65 |
| LDH (U/L), median (IQR) | 578 (451, 832) | 561 (413, 722) | 0.34 |
| C-reactive protein (mg/dL), median (IQR) | 15.8 (9.2, 22.9) | 21.1 (13.6, 28.9) | **0.03** |
| Procalcitonin (ng/mL), median (IQR) | 3.5 (1.2, 15.9) | 4.5 (1.6, 13.9) | 0.46 |
| **Day 3 (available values/total group)** | 52/57 | 40/48 |  |
| Leukocytes (/nL), median (IQR) | 16.5 (8.3, 20.3) | 14.1 (10.5, 17.4) | 0.61 |
| Haemoglobin (g/dL), median (IQR) | 8.2 (7.9, 8.8) | 8.2 (7.8, 8.7) | 0.74 |
| Platlets (/nL), median (IQR) | 144 (77, 195) | 118 (74, 193) | 0.44 |
| INR, median (IQR) | 1.1 (1.1, 1.4) | 1.1 (1.1, 1.3) | 0.96 |
| aPTT (sec), median (IQR) | 46.9 (40.5, 59.9) | 44.1 (38.0, 57.9) | 0.55 |
| Fibrinogen (mg/dL), median (IQR) | 512 (407, 673) | 534 (366, 629) | 0.83 |
| Serum creatinine (mg/dL), median (IQR) | 1.5 (0.8, 2.4) | 2.0 (0.9, 2.7) | 0.24 |
| Blood urea nitrogen (mg/dL), median (IQR) | 39 (27, 55) | 39.1 (33.1, 69.2) | 0.41 |
| eGFR (MDRD formula; mL/min/1.73m²), median (IQR) | 45 (30, 60) | 38 (27, 60) | 0.32 |
| Bilirubin (mg/dL), median (IQR) | 1.3 (0.9, 3.5) | 2.2 (1.1, 5.1) | 0.07 |
| Myoglobin (µg/L), median (IQR) | 949 (415, 3641) | 1246 (610, 3669) | 0.72 |
| LDH (U/L), median (IQR) | 604 (449, 812) | 510 (402, 691) | 0.19 |
| C-reactive protein (mg/dL), median (IQR) | 14.5 (8.9, 22.7) | 21.6 (11.0, 26.9) | **0.02** |
| Procalcitonin (ng/mL), median (IQR) | 4.7 (1.1, 13.0) | 2.6 (1.4, 8.9) | 0.83 |
| **Day 4 (available values/total group)** | 47/57 | 38/48 |  |
| Leukocytes (/nL), median (IQR) | 13.3 (8.9, 19.0) | 14.4 (10.8, 19.8) | 0.61 |
| Haemoglobin (g/dL), median (IQR) | 8.3 (8.0, 8.8) | 8.2 (7.6, 8.6) | 0.32 |
| Platlets (/nL), median (IQR) | 147 (83, 186) | 116 (61, 173) | 0.26 |
| INR, median (IQR) | 1.1 (1.0, 1.5) | 1.2 (1.1, 1.3) | 0.80 |
| aPTT (sec), median (IQR) | 45.8 (39.8, 56.0) | 42.9 (38.6, 50.4) | 0.19 |
| Fibrinogen (mg/dL), median (IQR) | 539.5 (443.0, 673.0) | 560.5 (414.5, 681.0) | 0.79 |
| Serum creatinine (mg/dL), median (IQR) | 1.5 (1.0, 2.6) | 1.8 (0.8, 2.6) | 0.75 |
| Blood urea nitrogen (mg/dL), median (IQR) | 43.9 (27.1, 58.7) | 41.5 (33.1, 68.7) | 0.71 |
| eGFR (MDRD formula; mL/min/1.73m²), median (IQR) | 47 (26, 60) | 37 (26, 60) | 0.60 |
| Bilirubin (mg/dL), median (IQR) | 1.3 (0.8, 2.9) | 2.2 (1.1, 5.3) | 0.08 |
| Myoglobin (µg/L), median (IQR) | 847 (288, 2859) | 1396 (505, 2556) | 0.58 |
| LDH (U/L), median (IQR) | 557 (440, 710) | 519 (447, 700) | 0.45 |
| C-reactive protein (mg/dL), median (IQR) | 14.2 (9.7, 20.4) | 21.1 (13.1, 25.1) | **0.03** |
| Procalcitonin (ng/mL), median (IQR) | 2.2 (0.8, 13.7) | 2.7 (1.3, 8.5) | 0.79 |
| **Day 5 (available values/total group)** | 46/57 | 37/48 |  |
| Leukocytes (/nL), median (IQR) | 13.7 (9.1, 19.4) | 12.7 (9.9, 18.9) | 0.90 |
| Haemoglobin (g/dL), median (IQR) | 8.4 (7.6, 8.9) | 8.6 (7.9, 9.1) | 0.67 |
| Platlets (/nL), median (IQR) | 145 (84, 186) | 101 (72, 167) | 0.14 |
| INR, median (IQR) | 1.2 (1.1, 1.4) | 1.2 (1.1, 1.3) | 0.81 |
| aPTT (sec), median (IQR) | 46.8 (40.8, 55.8) | 41.2 (36.4, 57.0) | 0.18 |
| Fibrinogen (mg/dL), median (IQR) | 624 (464, 736) | 643 (471, 832) | 0.63 |
| Serum creatinine (mg/dL), median (IQR) | 1.6 (1.0, 2.7) | 1.9 (0.8, 2.7) | 0.64 |
| Blood urea nitrogen (mg/dL), median (IQR) | 42.2 (28.2, 60.3) | 51.9 (34.0, 65.9) | 0.44 |
| eGFR (MDRD formula; mL/min/1.73m²), median (IQR) | 43 (25, 60) | 35 (25, 60) | 0.52 |
| Bilirubin (mg/dL), median (IQR) | 1.7 (0.8, 4.0) | 2.5 (1.2, 5.5) | **0.05** |
| Myoglobin (µg/L), median (IQR) | 907 (250, 3448) | 1184 (499, 3633) | 0.33 |
| LDH (U/L), median (IQR) | 593 (470, 791) | 534 (433, 708) | 0.39 |
| C-reactive protein (mg/dL), median (IQR) | 15.2 (11.8, 23.2) | 19.3 (14.4, 30.2) | **0.04** |
| Procalcitonin (ng/mL), median (IQR) | 2.1 (0.8, 15.5) | 3.9 (1.2, 9.7) | 0.70 |
| **Day 6 (available values/total group)** | 41/57 | 35/48 |  |
| Leukocytes (/nL), median (IQR) | 13 (10, 20) | 15 (10, 20) | 0.94 |
| Haemoglobin (g/dL), median (IQR) | 8.4 (7.8, 8.8) | 8.6 (8.1, 9.4) | 0.19 |
| Platlets (/nL), median (IQR) | 130 (83, 196) | 102 (70, 174) | 0.24 |
| INR, median (IQR) | 1.1 (1.1, 1.3) | 1.2 (1.1, 1.3) | 0.18 |
| aPTT (sec), median (IQR) | 46.2 (40.0; 56.0) | 48 (42.5; 54.9) | 0.54 |
| Fibrinogen (mg/dL), median (IQR) | 619 (439, 778) | 660 (460, 851) | 0.63 |
| Serum creatinine (mg/dL), median (IQR) | 1.7 (1.0, 2.6) | 1.9 (1.0, 2.6) | 0.67 |
| Blood urea nitrogen (mg/dL), median (IQR) | 46.1 (30.8, 61.1) | 51.4 (35.3, 64.4) | 0.39 |
| eGFR (MDRD formula; mL/min/1.73m²), median (IQR) | 38 (28, 60) | 38 (24, 60) | 0.62 |
| Bilirubin (mg/dL), median (IQR) | 1.5 (0.8, 4.0) | 2.9 (1.0, 7.3) | 0.14 |
| Myoglobin (µg/L), median (IQR) | 1036 (328, 3727) | 990 (454, 4760) | 0.48 |
| LDH (U/L), median (IQR) | 551 (453, 1016) | 533 (412, 647) | 0.46 |
| C-reactive protein (mg/dL), median (IQR) | 18.7 (12.7, 22.9) | 19.8 (13.5, 30.2) | 0.20 |
| Procalcitonin (ng/mL), median (IQR) | 2.8 (0.7, 9.1) | 3.8 (1.3, 14.6) | 0.53 |
| **Day 7 (available values/total group)** | 36/57 | 34/48 |  |
| Leukocytes (/nL), median (IQR) | 13.1 (10.6, 20) | 14.8 (10.6, 20.6) | 0.60 |
| Haemoglobin (g/dL), median (IQR) | 8.6 (8.0, 9.1) | 8.7 (8.3, 9.2) | 0.33 |
| Platlets (/nL), median (IQR) | 127 (87, 184) | 109 (74, 172) | 0.69 |
| INR, median (IQR) | 1.1 (1.1, 1.2) | 1.2 (1.1, 1.4) | 0.07 |
| aPTT (sec), median (IQR) | 45.0 (40.9, 57.0) | 45.9 (38.7, 57.2) | 0.93 |
| Fibrinogen (mg/dL), median (IQR) | 624 (456, 900) | 660 (418, 849) | 0.97 |
| Serum creatinine (mg/dL), median (IQR) | 1.9 (1.2, 2.5) | 1.8 (1.1, 2.7) | 0.84 |
| Blood urea nitrogen (mg/dL), median (IQR) | 44.1 (34.1, 65.5) | 50.7 (39.0, 70.6) | 0.44 |
| eGFR (MDRD formula; mL/min/1.73m²), median (IQR) | 37 (27, 60) | 36 (24, 60) | 0.78 |
| Bilirubin (mg/dL), median (IQR) | 1.7 (1.0, 5.1) | 3.5 (1.0, 11.1) | 0.28 |
| Myoglobin (µg/L), median (IQR) | 1084 (386, 3759) | 932 (526, 4356) | 0.82 |
| LDH (U/L), median (IQR) | 572 (433, 1073) | 617 (432, 722) | 0.90 |
| C-reactive protein (mg/dL), median (IQR) | 16.8 (10.3, 23.5) | 19.2 (12.6, 27.9) | 0.41 |
| Procalcitonin (ng/mL), median (IQR) | 3.3 (1.2, 12.0) | 3.4 (1.1, 14.5) | 0.99 |
| Activated partial thromboplastin time, aPTT; continuous renal replacement therapy, CRRT; day, d; extracorporeal membrane oxygenation, ECMO; estimated glomerular filtration rate, eGFR; international normalized ratio, INR; lactate dehydrogenase, LDH; liter, L; Modification of Diet in Renal Disease, MDRD; relative risk, RR; unit, U. | | | |

**Supplementary Table 2.** Seven-day follow-up of CRRT settings comparing integrated and parallel configurations of CRRT and ECMO.

| **Day after starting support** | **Integrated approach group n=57** | **Parallel approach group  n=48** | **p value** |
| --- | --- | --- | --- |
| **Day 1** |  |  |  |
| Blood flow (mL/min), median (IQR) | 100 (100, 100) | 100 (100, 100) | 0.46 |
| Access line pressure (mmHg), median (IQR) | 100 (68, 130) | -25 (-33, 83) | **<0.01** |
| Return line pressure (mmHg), median (IQR) | 160 (120, 190) | 50 (40, 143) | **<0.01** |
| Transmembrane pressure (mmHg), median (IQR) | 30 (20, 33) | 30 (20, 30) | 0.97 |
| Ultrafiltration rate (mL/h), median (IQR) | 400( 338, 413) | 400 (300, 400) | 0.58 |
| Dialysate flow (mL/h), median (IQR) | 1500 (1000, 1500) | 1500 (1000, 1813) | 0.42 |
| **Day 2** |  |  |  |
| Blood flow (mL/min), median (IQR) | 100 (100, 100) | 100 (100, 100) | 1 |
| Access line pressure (mmHg), median (IQR) | 100 (65, 120) | -20 (-30, 100) | **<0.01** |
| Return line pressure (mmHg), median (IQR) | 170 (130, 190) | 50 (40, 150) | **<0.01** |
| Transmembrane pressure (mmHg), median (IQR) | 30 (25, 40) | 30 (30, 40) | 0.68 |
| Ultrafiltration rate (mL/h), median (IQR) | 400 (300, 450) | 400 (300, 400) | 0.97 |
| Dialysate flow (mL/h), median (IQR) | 1750 (1500, 2000) | 1500 (1250, 1750) | 0.06 |
| **Day 3** |  |  |  |
| Blood flow (mL/min), median (IQR) | 100 (100, 100) | 100 (100, 100) | 1 |
| Access line pressure (mmHg), median (IQR) | 105 (50, 135) | -10 (-30, 85) | **<0.01** |
| Return line pressure (mmHg), median (IQR) | 160 (120, 200) | 50 (40, 150) | **<0.01** |
| Transmembrane pressure (mmHg), median (IQR) | 30 (28, 40) | 30 (30, 40) | 0.24 |
| Ultrafiltration rate (mL/h), median (IQR) | 400 (350, 450) | 400 (300, 413) | 0.56 |
| Dialysate flow (mL/h), median (IQR) | 1500 (1500, 1763) | 1500 (1388, 1813) | 0.85 |
| **Day 4** |  |  |  |
| Blood flow (mL/min), median (IQR) | 100 (100, 100) | 100 (100, 100) | 0.20 |
| Access line pressure (mmHg), median (IQR) | 80 (50, 120) | -10 (-20, 85) | **<0.01** |
| Return line pressure (mmHg), median (IQR) | 135 (103, 198) | 60 (40, 160) | **<0.01** |
| Transmembrane pressure (mmHg), median (IQR) | 30 (23, 40) | 30 (30, 40) | 0.89 |
| Ultrafiltration rate (mL/h), median (IQR) | 400 (350, 450) | 400 (400, 500) | 0.64 |
| Dialysate flow (mL/h), median (IQR) | 1500 (1500, 2000) | 1700 (1500, 2000) | 0.49 |
| **Day 5** |  |  |  |
| Blood flow (mL/min), median (IQR) | 100 (100, 100) | 100 (100, 100) | 0.44 |
| Access line pressure (mmHg), median (IQR) | 80 (40, 110) | -10 (-30, 100) | **0.02** |
| Return line pressure (mmHg), median (IQR) | 140 (100, 170) | 60 (50, 160) | **0.02** |
| Transmembrane pressure (mmHg), median (IQR) | 30 (30, 40) | 30 (30, 40) | 0.97 |
| Ultrafiltration rate (mL/h), median (IQR) | 400 (350, 500) | 400 (300, 500) | 0.18 |
| Dialysate flow (mL/h), median (IQR) | 1500 (1500, 1750) | 1500 (1250, 1750) | 0.48 |
| **Day 6** |  |  |  |
| Blood flow (mL/min), median (IQR) | 100 (100, 100) | 100 (100, 100) | 0.38 |
| Access line pressure (mmHg), median (IQR) | 90 (40, 120) | -20 (-40, 100) | **<0.01** |
| Return line pressure (mmHg), median (IQR) | 150 (90, 190) | 60 (40, 178) | **0.03** |
| Transmembrane pressure (mmHg), median (IQR) | 30 (30, 40) | 40 (23, 40) | 0.64 |
| Ultrafiltration rate (mL/h), median (IQR) | 400 (350, 450) | 400 (350, 400) | 0.53 |
| Dialysate flow (mL/h), median (IQR) | 1500 (1500, 1750) | 1500 (1250, 1750) | 0.30 |
| **Day 7** |  |  |  |
| Blood flow (mL/min), median (IQR) | 100 (100, 100) | 100 (100, 100) | 1 |
| Access line pressure (mmHg), median (IQR) | 80 (20, 130) | -20 (-30, 100) | **<0.01** |
| Return line pressure (mmHg), median (IQR) | 140 (100, 190) | 50 (40, 160) | **<0.01** |
| Transmembrane pressure (mmHg), median (IQR) | 40 (30, 40) | 30 (20, 40) | 0.14 |
| Ultrafiltration rate (mL/h), median (IQR) | 400 (300, 450) | 400 (300, 400) | 0.77 |
| Dialysate flow (mL/h), median (IQR) | 1500 (1250, 1750) | 1250 (1250, 1688) | 0.19 |
| Continuous renal replacement therapy, CRRT; day, d; extracorporeal membrane oxygenation, ECMO; liter, L; minute, min. | | | |

**Supplementary Table 3.** Seven-day follow-up of ECMO settings comparing integrated and parallel configurations of CRRT and ECMO.

| **Day after starting support** | **Integrated approach group n=57** | **Parallel approach group  n=48** | **p value** |
| --- | --- | --- | --- |
| **Day 1** |  |  |  |
| ECMO blood flow (L/min), median (IQR) | 4.4 (3.5, 5.2) | 4.3 (3.5, 5.5) | 0.55 |
| Return pressure (mmHg), median (IQR) | 144 ( 106, 170) | 170 (117, 207) | 0.09 |
| Drainage pressure (mmHg), median (IQR) | -74 (-114, -46) | -70 (-121, -44) | 0.75 |
| **Day 2** |  |  |  |
| ECMO blood flow (L/min), median (IQR) | 4.4 (3.5, 5.1) | 4.5 (3.5, 5.8) | 0.25 |
| Return pressure (mmHg), median (IQR) | 144 (111, 166) | 166 (136, 201) | 0.10 |
| Drainage pressure (mmHg), median (IQR) | -72 (-101, -41) | -78 (-110, -53) | 0.37 |
| **Day 3** |  |  |  |
| ECMO blood flow (L/min), median (IQR) | 4.6 (3.6, 5.3) | 4.6 (3.5, 5.8) | 0.68 |
| Return pressure (mmHg), median (IQR) | 145 (120, 198) | 169 (116, 212) | 0.37 |
| Drainage pressure (mmHg), median (IQR) | -71 (-103, -44) | -76 (-107, -41) | 0.90 |
| **Day 4** |  |  |  |
| ECMO blood flow (L/min), median (IQR) | 4.2 (3.3, 5.0) | 4.6 (3.4, 5.8) | 0.22 |
| Return pressure (mmHg), median (IQR) | 136 (107, 188) | 165 (122, 212) | 0.19 |
| Drainage pressure (mmHg), median (IQR) | -62 (-91, -40) | -78 (-124, -44) | 0.19 |
| **Day 5** |  |  |  |
| ECMO blood flow (L/min), median (IQR) | 4.4 (3.2, 5.3) | 4.4 (2.8, 5.3) | 0.94 |
| Return pressure (mmHg), median (IQR) | 132 (109, 190) | 153 (116, 181) | 0.47 |
| Drainage pressure (mmHg), median (IQR) | -72 (-97, -46) | -76 (-113, -39) | 0.69 |
| **Day 6** |  |  |  |
| ECMO blood flow (L/min), median (IQR) | 4.5 (3.2, 5.2) | 4.5 (3.3, 5.3) | 0.91 |
| Return pressure (mmHg), median (IQR) | 141 (111, 187) | 156 (106, 190) | 0.63 |
| Drainage pressure (mmHg), median (IQR) | -68 (-93, -43) | -85 (-107, -31) | 0.68 |
| **Day 7** |  |  |  |
| ECMO blood flow (L/min), median (IQR) | 4.6 (2.9, 5.0) | 4.6 (3.2, 5.4) | 0.44 |
| Return pressure (mmHg), median (IQR) | 137 (92, 177) | 155 (132, 190) | 0.30 |
| Drainage pressure (mmHg), median (IQR) | -72 (-87, -36) | -88 (-112, -33) | 0.26 |
| Continuous renal replacement therapy, CRRT; day, d; extracorporeal membrane oxygenation, ECMO; liter, L; minute, min. | | | |

1. **Supplementary Figures**

**Supplementary Figure 1.**  Laboratory values over the first seven days in patients with integrated (n=57) or parallel (n=48) configurations of CRRT and ECMO.

Activated partial thromboplastin time, aPTT; continuous renal replacement therapy, CRRT; day, d; extracorporeal membrane oxygenation, ECMO; estimated glomerular filtration rate, eGFR; international normalized ratio, INR; lactate dehydrogenase, LDH; liter, L; unit, U.





**Supplementary Figure 2.** CRRT (A) and ECMO (B) settings over seven days with integrated (n=57) or parallel (n=48) configuration.

Continuous renal replacement therapy, CRRT; extracorporeal membrane oxygenation, ECMO.





**Supplementary Figure 3.** Correlation between return ECMO pressures and CRTT return line pressures in integrated and parallel configurations. Pressure levels of the return pressure in the ECMO system and the return line in the CRRT circuit are shown for the 57 patients treated with the integrated configuration of ECMO and CRRT (A) and for the 48 patients treated with the parallel configuration of ECMO and CRRT (B). Pressure levels are correlated at different time points: at the start of support, during the first seven days and at the end of support.

Continuous renal replacement therapy, CRRT; extracorporeal membrane oxygenation, ECMO.
